# Radiologically Probable Late-Onset Von Hippel–Lindau Syndrome: A Case Report

**DOI:** 10.7759/cureus.110423

**Published:** 2026-06-07

**Authors:** Venkatesh Y, Bheru Dan Charan, Hemanth Kumar G R, Tarikjot Singh, Archit Gupta

**Affiliations:** 1 Department of Radiodiagnosis and Interventional Radiology, All India Institute of Medical Sciences Rishikesh, Rishikesh, IND; 2 Department of Neuroradiology, All India Institute of Medical Sciences, New Delhi, New Delhi, IND

**Keywords:** pancreatic cystosis, pancreatic neuroendocrine tumor, pheochromocytoma, renal cell carcinoma, serous cystadenoma, von hippel-lindau syndrome (vhl)

## Abstract

Von Hippel-Lindau (VHL) syndrome is a rare hereditary tumor syndrome characterized by multisystem involvement and variable clinical presentation. Radiological evaluation plays a pivotal role in early diagnosis and surveillance, particularly in settings where histopathological and genetic confirmation may not be feasible.

We report a case of a 69-year-old male presenting with nonspecific abdominal complaints. Triple-phase contrast-enhanced CT of the thorax and abdomen demonstrated diffuse pancreatic cystosis, multiple hypervascular pancreatic lesions favoring pancreatic neuroendocrine tumors, serous cystadenomas, a hypervascular left adrenal lesion suspicious for pheochromocytoma, and multifocal left renal cortical masses suspicious for renal cell carcinoma. Based on the characteristic constellation of multiorgan thoracoabdominal imaging findings, the case was interpreted as radiologically probable late-onset VHL syndrome. Histopathological, biochemical, ophthalmologic, central nervous system (CNS) imaging, and genetic confirmation could not be obtained because the patient was lost to follow-up.

## Introduction

Von Hippel-Lindau (VHL) syndrome is a rare autosomal dominant hereditary neoplastic syndrome caused by mutations involving the VHL tumor suppressor gene located on chromosome 3p25-26 [1]. The VHL gene functions as a tumor suppressor gene, and its inactivation promotes angiogenesis and tumor formation through dysregulation of hypoxia-related pathways [1].

The disease is characterized by predisposition to multiple benign and malignant tumors involving the central nervous system (CNS), retina, kidneys, pancreas, adrenal glands, and reproductive organs [1,2]. The most common manifestations of VHL disease include retinal and CNS hemangioblastomas, clear cell renal cell carcinoma (RCC), pheochromocytoma, pancreatic cysts, serous cystadenomas, pancreatic neuroendocrine tumors (PNETs), and endolymphatic sac tumors [1,2].

The estimated incidence of VHL disease is approximately 1 in 36,000-50,000 live births with high age-dependent penetrance. VHL syndrome typically manifests during the second to fourth decades of life, with most affected individuals developing characteristic manifestations by early adulthood and more than 90% demonstrating disease penetrance by the sixth decade of life [2,3]. Approximately 20% of patients develop VHL disease due to de novo mutations without a positive family history [3]. CNS hemangioblastomas are considered the hallmark lesions and remain one of the major causes of morbidity and mortality in affected patients [4].

Pancreatic manifestations are frequently encountered in VHL disease and include simple pancreatic cysts, diffuse pancreatic cystosis, serous cystadenomas, and pancreatic neuroendocrine tumors [5]. VHL-associated pancreatic neuroendocrine tumors are usually nonfunctional and exhibit lower metastatic potential compared with sporadic neuroendocrine tumors [5]. Presentation in the seventh decade is distinctly uncommon and may delay recognition because manifestations can be mistaken for sporadic neoplastic processes rather than an underlying hereditary tumor syndrome [6,7].

Recognition of characteristic imaging findings is essential because radiological evaluation plays a pivotal role in diagnosis, surveillance, and management planning, especially in settings where genetic testing may not be readily available.

We report a rare case of radiologically probable late-onset Von Hippel-Lindau syndrome in a 69-year-old male presenting with diffuse pancreatic cystosis, multiple hypervascular pancreatic lesions, serous cystadenomas, an adrenal lesion suspicious for pheochromocytoma, and multifocal renal cortical masses suspicious for renal cell carcinoma. The diagnosis was based predominantly on the characteristic constellation of imaging findings in the absence of histopathological, biochemical, ophthalmologic, CNS imaging, and genetic confirmation.

## Case presentation

A 69-year-old male presented with intermittent, non-specific abdominal pain and fatigue for one month. The patient denied headache, seizures, visual disturbances, gait imbalance, focal neurological deficits, hearing loss, or other symptoms suggestive of CNS involvement at presentation. There was no known family history of hereditary tumor syndromes. Physical examination was unremarkable. Routine laboratory parameters, including renal and liver function tests, were within normal limits. Serum sodium was mildly reduced (132 mmol/L). Serum ferritin was markedly elevated (1175.9 ng/mL) with low serum iron levels (25.6 µg/dL), findings that may reflect an underlying inflammatory or chronic disease process. Triphasic contrast-enhanced CT of the thorax and abdomen was performed. The pancreas was diffusely enlarged and replaced by numerous variable-sized cystic lesions involving the head, body and tail, consistent with pancreatic cystosis (Figure 1A-1B). Three well-defined hypervascular solid lesions were identified within the pancreas. These demonstrated intense enhancement in the arterial phase with relative reduction in enhancement during the portal venous phase and persistent mild enhancement on delayed phase imaging, favoring hypervascular pancreatic lesions. Lesion characterization was based on findings from arterial, portal venous, and delayed phase acquisitions of the triple-phase CT examination (Figure 1A-1B).

**Figure 1:**
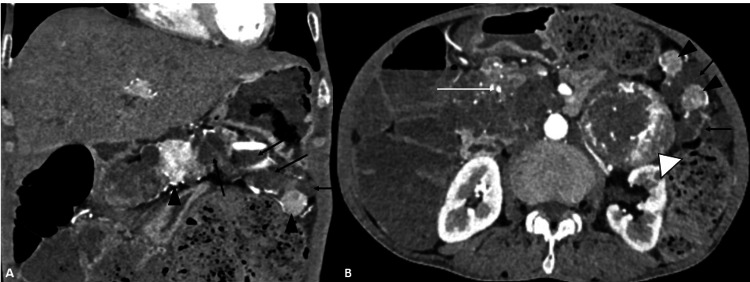
Coronal and axial post-contrast late arterial phase computed tomography images of the abdomen (A) Coronal reformatted image shows two avidly enhancing solid lesions (black arrowheads) in the body and tail of the pancreas, consistent with hypervascular pancreatic lesions. The remaining pancreas is replaced by numerous variable-sized cystic lesions (black arrows), suggestive of pancreatic cystosis. (B) Axial image demonstrates a large lobulated microcystic lesion with enhancing septations and calcific foci (white arrow) in the pancreatic head, suggestive of serous cystadenoma. Two avidly enhancing solid lesions (black arrowheads) are again seen in the pancreatic tail, consistent with hypervascular pancreatic lesions. A well-circumscribed heterogeneously enhancing lesion in the left suprarenal region (white arrowhead) represents a hyper vascular adrenal lesion suspicious for pheochromocytoma.

In addition, three lobulated microcystic lesions with enhancing septations were seen, consistent with serous cystadenomas (Figure 2A-2B).

**Figure 2:**
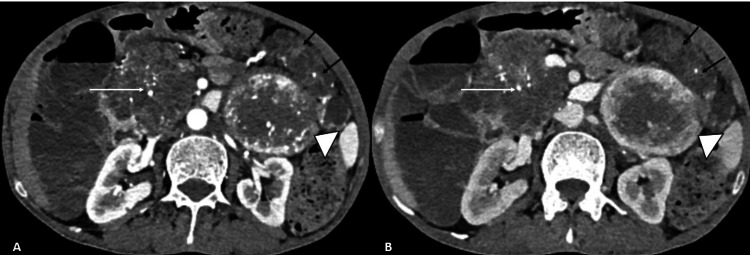
Axial post-contrast computed tomography images of the abdomen (A) Late arterial phase image demonstrates a large lobulated microcystic lesion with enhancing septations and calcific foci (white arrow) in the pancreatic head. Two additional smaller lesions with similar microcystic morphology are seen in the pancreatic tail (black arrows). (B) Portal venous phase image demonstrates persistent enhancement of the septations without appreciable washout, findings suggestive of serous cystadenomas. An arterial phase avidly enhancing well-circumscribed, predominantly cystic lesion in the left suprarenal region (white arrowhead) demonstrates relative reduction in enhancement on portal venous phase imaging, raising suspicion for pheochromocytoma. However, adrenal metastasis could not be completely excluded in the absence of biochemical and histopathological confirmation.

A well-circumscribed predominantly cystic heterogeneously enhancing lesion was noted in the left adrenal gland, demonstrating avid arterial enhancement with relative reduction in enhancement on portal venous and delayed phase images, raising suspicion for pheochromocytoma. However, hypervascular adrenal metastasis remained an important differential consideration in the absence of biochemical and histopathological confirmation (Figure 3A-3B).

**Figure 3:**
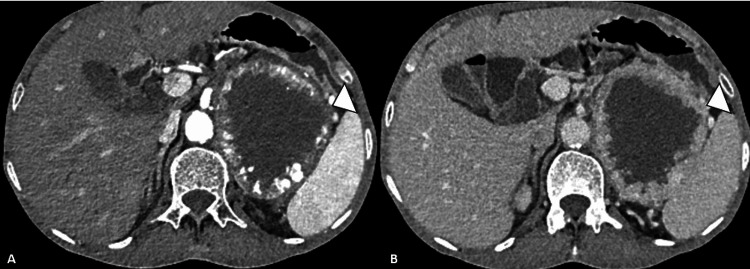
Axial post-contrast computed tomography images of the abdomen (A) Late arterial phase image demonstrates an avidly enhancing, well-circumscribed predominantly cystic lesion (white arrow head) in the left suprarenal region. (B) Portal venous phase image demonstrates a relative reduction in enhancement of the lesion compared with the arterial phase. The imaging findings raise suspicion for a hypervascular adrenal lesion, like pheochromocytoma; however, adrenal metastasis could not be completely excluded in the absence of biochemical and histopathological confirmation.

Two heterogeneously enhancing cortical lesions were present in the left kidney, demonstrating arterial phase hyperenhancement with relative reduction in enhancement on portal venous and delayed phase images, raising suspicion for multifocal renal cell carcinoma (Figure 4A-4C).

**Figure 4:**
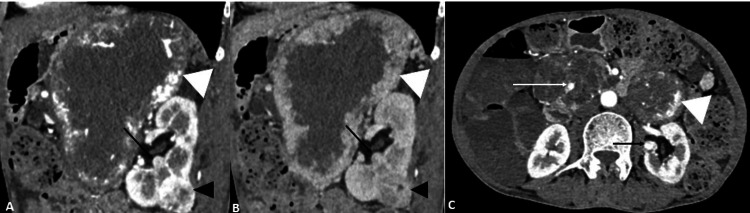
Sagittal and axial post-contrast computed tomography images of the abdomen (A) Sagittal reformatted late arterial phase image demonstrates an avidly enhancing partially exophytic solid lesion (black arrowhead) in the lower pole of the left kidney. Another tiny exophytic lesion with similar enhancement characteristics is also noted in the lower pole of the left kidney (black arrow). (B) Sagittal reformatted portal venous phase image demonstrates relative reduction in enhancement of the cortical lesions compared with the arterial phase, raising suspicion for multifocal renal cell carcinoma. (C) Axial late arterial phase demonstrates more clearly the tiny avidly enhancing partially exophytic solid lesion (black arrow) in the lower pole of the left kidney. An avidly enhancing, well-circumscribed predominantly cystic lesion in the left suprarenal region (white arrowhead) demonstrates a relative reduction in enhancement on portal venous phase images, raising suspicion for pheochromocytoma. However, hypervascular adrenal metastasis could not be completely excluded in the absence of biochemical and histopathological confirmation. A large lobulated microcystic lesion with enhancing septations and calcific foci (white arrow) in the pancreatic head region consistent with serous cystadenoma.

As part of the staging evaluation, contrast-enhanced CT of the thorax was performed during the same sitting as the triple-phase CT examination of the abdomen. No radiologically evident thoracic metastases or other significant thoracic abnormalities were identified. However, dedicated whole-body staging investigations such as PET-CT, somatostatin receptor imaging, bone scintigraphy, or MRI were not performed because the patient was lost to follow-up after the initial imaging evaluation. In the absence of histopathological and genetic confirmation, the constellation of multiorgan thoracoabdominal imaging findings favored a diagnosis of radiologically probable late-onset VHL syndrome.

Biochemical evaluation for catecholamine excess, including plasma metanephrines and urinary vanillylmandelic acid levels, was planned because of the adrenal lesion suspicious for pheochromocytoma; however, these investigations could not be completed because the patient was lost to follow-up. Multidisciplinary evaluation, including endocrinology, urology, oncology, ophthalmologic screening, CNS imaging, and genetic counseling, was advised. Surgical and surveillance-based management options were discussed with the patient; however, further evaluation and management could not be undertaken because the patient was lost to follow-up.

## Discussion

Diffuse pancreatic cystosis is relatively uncommon but highly suggestive of VHL syndrome when associated with other characteristic manifestations. Pancreatic neuroendocrine tumors associated with VHL are typically hypervascular and nonfunctional [5]. The pancreatic lesions identified in our patient demonstrated imaging characteristics suggestive of pancreatic serous cystadenomas, while the arterial phase hyperenhancing lesions favored pancreatic neuroendocrine tumors. Differential considerations included hypervascular pancreatic metastases from probable renal cell carcinoma, which may closely mimic pancreatic neuroendocrine tumors on contrast-enhanced imaging. However, the coexistence of diffuse pancreatic cystosis, serous cystadenomas, multiplicity of pancreatic lesions, and the overall imaging pattern favored pancreatic neuroendocrine tumors. Nevertheless, definitive differentiation could not be established in the absence of histopathological confirmation.

Renal manifestations are among the most important determinants of prognosis in VHL syndrome because renal cell carcinoma remains a leading cause of disease-related mortality [6]. Multifocal renal cortical masses in our patient were highly suspicious for RCC. The left adrenal lesion demonstrated avid arterial enhancement with relative reduction in enhancement on portal venous and delayed phase imaging, raising suspicion for pheochromocytoma, another recognized manifestation of VHL syndrome [8]. However, hypervascular adrenal metastasis remained an important differential diagnosis, and definitive differentiation could not be established without biochemical evaluation, histopathological confirmation, or dedicated adrenal MRI.

The diagnosis of VHL syndrome is established using a combination of clinical manifestations, family history, and genetic testing [6,8]. In the present case, complete systemic screening, histopathological confirmation, biochemical evaluation, ophthalmologic assessment, CNS imaging, and genetic testing were unavailable because the patient was lost to follow-up. Therefore, the diagnosis should be interpreted as radiologically probable rather than definitive.

Other differential considerations included multiple endocrine neoplasia type 1 (MEN1), sporadic multifocal pancreatic neuroendocrine tumors, and other hereditary renal tumor syndromes. MEN1 commonly presents with multiple pancreatic neuroendocrine tumors but is less frequently associated with diffuse pancreatic cystosis, serous cystadenomas, pheochromocytoma, and multifocal renal masses. The coexistence of these lesions therefore favored VHL syndrome over alternative hereditary or sporadic neoplastic conditions [1,2,5].

Radiological imaging plays a central role in the diagnosis and surveillance of VHL syndrome. MRI remains the preferred modality for evaluation of CNS lesions, whereas multiphasic CT and MRI are useful for assessment of abdominal manifestations [6,8]. MRI was planned for further lesion characterization, but could not be performed because the patient was lost to follow-up. Triple-phase contrast-enhanced CT of the thorax and abdomen did not demonstrate radiologically evident distant metastatic disease within the scanned field of view; however, dedicated whole-body staging investigations and follow-up imaging were unavailable, and occult metastatic disease therefore cannot be completely excluded.

This case has several important limitations, including the absence of histopathological confirmation, biochemical endocrine evaluation, ophthalmologic assessment, CNS imaging, dedicated whole-body staging investigations, and genetic testing. Consequently, definitive differentiation between pancreatic neuroendocrine tumors and hypervascular pancreatic metastases, as well as between pheochromocytoma and adrenal metastatic disease, could not be established. Accordingly, all lesions should be interpreted as radiologically suspicious or probable rather than confirmed.

This case highlights an unusual late-onset presentation of VHL syndrome suggested by characteristic thoracoabdominal imaging findings and extensive pancreatic involvement. Recognition of characteristic thoracoabdominal imaging findings is crucial for early diagnosis, surveillance, multidisciplinary management and consideration of hereditary tumor syndromes, particularly when histopathological or genetic confirmation is unavailable.

## Conclusions

This case highlights the importance of recognizing characteristic multiorgan imaging findings that may suggest an underlying hereditary tumor syndrome such as VHL syndrome. Although a definitive diagnosis could not be established because of the absence of histopathological, biochemical, ophthalmologic, CNS imaging, and genetic confirmation, the constellation of imaging findings raised strong suspicion for radiologically probable late-onset VHL syndrome. Early recognition may facilitate timely multidisciplinary evaluation, surveillance, genetic counseling, and confirmation through additional investigations.
